# Smartphone Use Type, Fear of Missing Out, Social Support, and Smartphone Screen Time Among Adolescents in Korea: Interactive Effects

**DOI:** 10.3389/fpubh.2022.822741

**Published:** 2022-03-16

**Authors:** Hye-Young Song, Ji-Hye Kim

**Affiliations:** College of Nursing, Woosuk University, Chonbuk, South Korea

**Keywords:** adolescent, smartphone, ecological model, fear of missing out, social support

## Abstract

This study aims to examine the relationship between intrapersonal factors, interpersonal factors, smartphone screen time, and the moderating roles of interpersonal factors, on the basis of the ecological model. This study is a cross-sectional and descriptive study. A total of 428 participants from four public middle schools were selected through convenience sampling (55.1% female; Mean age 13.0 ± 0.78). Data were collected through self-report questionnaires that contained questions about sociodemographic characteristics, intrapersonal factors (types of smartphone use, Fear of missing out—FoMO), interpersonal factors (support from parents, teachers, and peers), and smartphone screen time. The collected data were analysed using descriptive statistics, *t*-test, ANOVA, Pearson's correlation coefficients, and hierarchical regression. The daily smartphone screen time was 4.05 ± 2.16 h. Results showed that social media (β = 0.155), games (β = 0.140), and FoMO (β = 0.227) were positively associated with smartphone screen time, while educational videos (β = −0.130) and parental support (β = −0.212) were negatively associated with smartphone screen time. Peers support moderated the association between games and smartphone screen time. Parental support moderated the association between educational videos, videos/movies/TV, and smartphone screen time. The findings highlight the direct and interactive roles of intrapersonal and interpersonal factors in predicting adolescents' smartphone screen time. Based on this study, the intrapersonal and interpersonal factors of adolescents should be comprehensively considered to intervene in their proper smartphone use.

## Introduction

Smartphones have become an inseparable part of our daily lives. The reason would be the usage of smartphone going beyond routine calls, allowing one to enjoy games, online-shopping, various social interacting activities and administrative work anytime, anywhere. This has brought us a convenient life, but excessive use of smartphones causes severe psychological, mental, and social problems as well (1–4).

According to the 2020 smartphone overdependence status survey, 35.8% of Korean adolescents were excessively dependent on smartphones with middle school students being the most dependent group at 39.6% (5). On average, Korean adolescents spend 4.8 h on smartphones on a daily basis for various reasons such as recreational activities, including games, videos, music, e-books, web-cartoons, social interactions *via* messengers, social media, or email, and education (6). As issues caused by smartphone overuse are expected to worsen, social interest and intervention are required.

Smartphone overdependence among adolescents could be driven by their developmental characteristics. Adolescents go through various physical, psychological, and societal changes, with which social demand towards them can be a source of stress (7). Furthermore, adolescents who have unstable emotions and lack self-control at times tend to resolve their concerns and issues by using smartphones (8). Adolescents use smartphones to avoid a variety of difficulties faced by them, and the pleasure coming from its use can lead to problematic smartphone use.

Numerous studies have been conducted on physical and mental health outcomes as well as academic achievement associated with problematic smartphone use. However, there is a dearth of research on the antecedents of such problematic smartphone use (9), and they mainly focus on socio-demographic and psychological characteristics. For example, gender (10), family income (10), depression (11), anxiety (11), impulsiveness (12), stress (11), and personality (13) were found to be factors associated with problematic smartphone use of adolescents. Hence, this study aims to contribute to the studies by identifying associable factors to adolescents' smartphone use, looking beyond the personal level, confirmed in previous studies, such as gender, economic status, and depression.

According to the ecological model, health behaviours are determined by intrapersonal, interpersonal, organisational, community, physical environmental, and policy levels (14, 15). Intrapersonal level refers to “characteristics of individual such as knowledge, attitudes, behaviour, self-concept, and skills” (16). Interpersonal level refers to “formal and informal social network and social systems, including the family, work group, and friendship networks” (16). Organisational level refers to the “formal or informal rules and influences of an organisation that affect individuals” (16). Community and policy level refers to the “organisation, institution, system, law, and policy of the community to which an individual belongs” (16). The interaction of factors at different levels influence behaviour and multi-level interventions should be most effective in changing behaviour (15). From the ecological viewpoint, factors that affect the health behaviours of adolescents can be found in various environments, and they do not independently influence their behaviour and development (17). In short, the structure and environments that affect adolescents' behaviour and development are in continuous interaction. Hence, interaction among major environmental factors of adolescents such as family, school, and friends, as well as intrapersonal factors affect individuals in various ways (7). The number of studies on how the contextual factors of children and adolescents influence various externalising behaviours and internalising problems based on such a theoretical framework is increasing (18–20). Just like other behaviours, adolescents' problematic smartphone use should be understood from an ecological perspective or a multi-level framework.

This study focused on the types of smartphone use and fear of missing out (FoMO) looking at intrapersonal factors of adolescents' problematic smartphone use. In this study, the type of smartphone use was regarded as behavioural characteristic that affects problematic smartphone use behaviour, and this was included as an intrapersonal level factor. As their excessive time spent on the Internet is associated with the type of Internet use, it can be assumed that problematic smartphone use is associated with the type of smartphone use (21). Recent studies reported that smartphone use for entertainment and games is associated with problematic smartphone use among adolescents (6, 22, 23). However, each study showed different results in regard to the type of smartphone use related to problematic smartphone use, and their focus was not on middle school students—the age group with the greatest concern. Also, there is not enough research that compares relative effects between various content consumption and problematic smartphone use. Hence, this study will compare which smartphone use type better predicts problematic smartphone use, hypothesising that the relationship between the middle school students' smartphone use type and smartphone screen time would be different.

Recently, FoMO has risen as an important factor that explains problematic smartphone use of adults. FoMO is the fear that one is missing out on information or trends, or the concern they are not included in peer culture, leading to constant need for checking what others are doing (24). Deficiency of basic psychological needs such as autonomy, competence, and relatedness can result in FoMO (24). Also, unmet needs for relatedness can generate fears of being socially excluded (25). A high level of FoMO is also associated with serious anxiety (26), low self-esteem (27) and low satisfaction of life (28). FoMO can be a particularly important factor for adolescents. For them, activities on social media through smartphones are a medium for freely expressing themselves and a process to establish social identity (6). As they fear being left behind from social relationships with peers, adolescents spend much time on social media, feeling stressed at the same time (29). Considering developmental characteristics of adolescent, it is meaningful to pay continued attention to their FoMO. Previous studies have reported that high FoMO is associated with excessive use of social media and PSU (26, 27). Therefore, this study hypothesised that a higher FoMO score of adolescents leads to a longer time spent on their smartphones.

This study focused on the support from parents, teachers and peers, as interpersonal factors of adolescents' problematic smartphone use. As they live within a limited environment, such as their family and school, parents, teachers, and peers form an important environment, being a social supporter as well (20). Many previous studies have shown that support from parents, teachers, and peers has a protective effect on the problematic behaviours of adolescents through direct and buffering effects (19, 20, 30). Adolescents recognise support from their parents, teachers, and peer, so they experience a high level of psychological well-being with improved self-control, ultimately decreasing risks of problematic behaviours (20). Hence, this study supposed that those with greater support from their parents, teachers, and peers—interpersonal level factors—will spend shorter time on smartphone.

As mentioned earlier, factors of various levels interact with each other, influencing adolescent behaviours (7). Previous research on problematic behaviours of adolescents have confirmed that social support works as a buffer (31), while being an environmental, protective factor that alleviates psychological problems such as depression, anxiety, and stress (32). Hence, it is assumed that there will be an interactive effect between personal factors and support from parents, teachers, and peers, in problematic smartphone use as well. This study hypothesised that the interaction between intrapersonal and interpersonal factors would affect the smartphone screen time.

**Figure 1:**
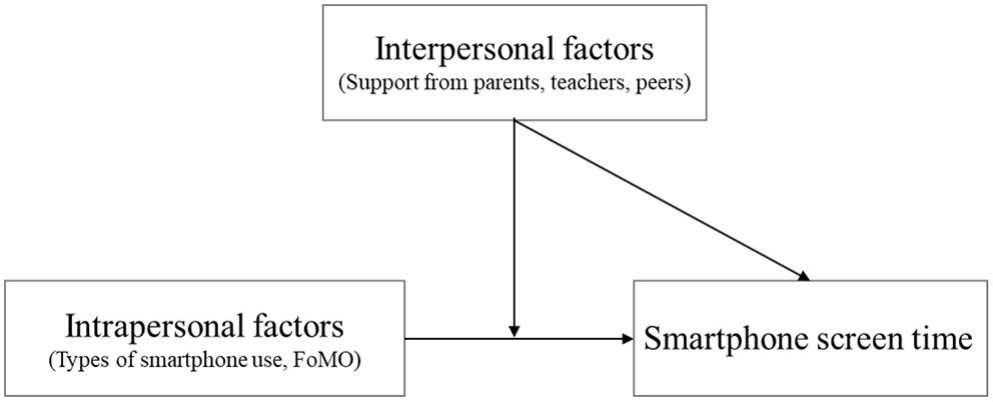
Theoretical models.

This study aims to investigate the intrapersonal and interpersonal factors of smartphone screen time among middle school students in Korea, on the basis of the ecological model (16). The interactive effects of intrapersonal and interpersonal factors are shown in Figure 1. In this study, smartphone screen time is a dependent variable —how much adolescents use their smartphones per day is one of the clearest determinants to problematic smartphone use (6). Analysis of all these comprehensive factors will help develop intervention strategies to address smartphone overuse issues among middle school students, while providing basic data to devise the guidelines for them to properly use smartphones.

## Materials and Methods

### Study Design

This study is a cross-sectional and secondary descriptive study to investigate the factors associated with smartphone screen time among middle school students in Korea, using an original survey data (33).

### Participants and Procedure

Students from four public middle schools in four Korean cities (Seoul, Gwangmyeong, Busan, and Cheongju) were selected through convenience sampling. Adolescents who were smartphone users, aged between 12 and 18, were recruited after consent to participate was obtained from them and their legal guardians. Middle school students who had received medical counselling or therapy for their psychological issues for the past year at medical institutions were excluded.

The data for this study were collected between 26 November and 20 December 2018. Survey instructions, consent forms and survey questionnaires written in Korean were distributed in a sealed envelope to students and their guardians. When they agreed to participate in the study, they were asked to sign the consent form and then answer the questionnaires. They were requested to seal them in the envelope after the completion of survey. The answered questionnaires were collected by the researcher for 2 days after the distribution.

The sample size was 0.15 which is a medium effect size of regression analysis, and the significance level of the two-tailed test was 0.05 with a statistical power of 0.95 (34). The minimum sample size came out as 208 by using G^*^Power software with 17 predictor variables. For this study, the questionnaires were distributed to 700 people and were retrieved from 553. Except for the 125 questionnaires with missing values in major variables, 428 questionnaires were used for the final analysis. The average age of the participants was 13.0 ± 0.78 (range: 12–15 years), and 236 participants (55.1%) were female.

The study was conducted according to the guidelines of the Declaration of Helsinki, and approved by the Institutional Review Board of H University Institutional Review Boards (Approval No. HYI-18-140-1) in the primary study (33). This study was exempted from ethical approval (Approval No. WS-2021-23).

### Measures

#### Socio-Demographic Characteristics

Gender, age, family structure, employment status of parents, subjective economic level, and health status were included. For the family structure, “Who do you currently live with”? was asked, and it was classified into “Double-parent family” and “Single parent family/No parent family.” For employment status of parents, “Are your parents employed”? was asked, and it was classified into “Both parents working” and “Single parent working/No working parent.” One item (“Please judge your family economic status in the following scale”) was used to understand the subjective economic level. Participants were asked to judge his or her family status using a 5-point scale (1 = “Extremely high” to 5 = “Extremely low”), and it was classified into “high,” “intermediate,” and “low.” For the subjective health status, “How do you feel about your health compared to your peers?” was asked.

#### Intrapersonal Factors

##### The Types of Smartphone Use

The items of types of smartphone use, including messengers, social media, games, videos/movies/TV, information-searching/web-surfing, and educational videos were adopted from Bea (22) and Jeong et al. (23). These questions were measured using a five-point Likert scale (1 = never, 5 = very often), to respond to the following question: “How often do you usually use your smartphone for each type of content?”

##### Fear of Missing Out

The single item FoMO scale, developed by Riordan et al. (35), was used to measure the FoMO level. It showed good concurrent validity, construct validity, and test–retest reliability (35). The question given to participants was “Do you experience the fear of missing out?”, using a 5-point Likert scale (1 = not true of me, 5 = extremely true of me). This shows that higher the score, higher the fears a participant has.

#### Interpersonal Factors

##### Parental Support

In order to measure how adolescents recognise the support from their parents, the Student Social Support Scale (SSSS) developed by Nolten (36) and adapted by Kim (37) was used. This instrument is used to measure the awareness of adolescents about how much they receive emotional, informational, evaluative, and material support from their parents (For example: “My parents are interested in me,” “My parents help me make decisions”). Consisting of 15 items, the questionnaire was based on a 5-point Likert scale (1 = strongly disagree, 5 = strongly agree). This means that higher the scores, the more likely they are to perceive they have received a high level of parental support. Cronbach's α in the developmental study (36) was 0.97, while Cronbach's α in the study was 0.947.

##### Teachers' Support

The Social Support Scale-Teacher developed by Kim (38) was used to measure adolescents' perception on support from their teachers. The instrument is used to measure perception among adolescents on the level of support, attention, recognition, and encouragement they have obtained from teachers, consisting of eight items in the questionnaire (For example: “My teacher helps me well,” “The teacher seems to recognise me as an important person”). All the question items were based on 5-point Likert scale (1 = strongly disagree, 5 = strongly agree). The highest score means the participants have the highest level of support from their teachers as perceived by them. Cronbach's α in the developmental study was 0.99 (38), while Cronbach's α in the study was 0.880.

##### Peers Support

The Social Support Scale-Peer developed by Kim (38) was used to measure adolescents' perception on support from their peers. The measurement helps identify the perception among adolescents on support, attention and encouragement, with eight question items (For example: “My friends seem to understand me well,” “My friends seem to like being with me”). All the question items were based on 5-point Likert scale (1 = strongly disagree, 5 = strongly agree). If the combined scores were high, it meant the participants receive high levels of support. Cronbach's α in the developmental study was 0.980 (38), while Cronbach's α in the study was 0.927.

#### Smartphone Screen Time

A single question item “How many hours do you spend on your phones a day?” was presented to participants. They recorded their daily screen time.

### Statistical Analysis

The variables were analysed with descriptive statistics. *T*-test, ANOVA and Scheffé test were conducted to examine the differences in the smartphone screen time by socio-demographic characteristics. Pearson's correlation coefficient was used to analyse the relationship between the intrapersonal factors, interpersonal factors, and smartphone screen time. To identify the factors associated with smartphone screen time, hierarchical regression analysis was conducted. Variables were entered into the models in three steps: covariates and intrapersonal factors (messengers, social media, games, videos/movies/TV, information-searching/web-surfing and educational videos, and FoMO) were simultaneously entered in Step 1, interpersonal factors (parental, teachers, and peers support) were entered in Step 2, and the interaction terms of intrapersonal factors and interpersonal factors were entered in Step 3. All continuous independent variables were standardised before the examination of the interactive effect. The statistical significance level was set at *p* < 0.05. The SPSS/WIN 25.0 software program was used for data analysis.

## Results

### Socio-Demographic Characteristics, Intrapersonal and Interpersonal Factors, and Smartphone Screen Time

The average age of the participants was 13.0 ± 0.78 (range: 12~15 years) and 236 participants (55.1%) were female. Most of the participants (84.6%) were from double-parent families. While 61.2% of participants had two working parents, 306 of them (71.5%) responded that their subjective economic level was intermediate (Table 1).

**Table 1:** Smartphone screen time by socio-demographic characteristics (*N* = 428).

**Characteristics**	**Categories**	***N***	**%**	**Smartphone screen time**	***t* or *F* (*p*)**
				**M ±SD**	
Gender	Male	192	44.9	3.48 ± 1.95	−5.031 (<0.001)
	Female	236	55.1	4.51 ± 2.21	
Age (year)	12^a^	128	29.9	3.43 ± 2.01	7.931 (0.001)
	13^b^	172	40.2	4.26 ± 2.20	a < b, c
	≥14^c^	128	29.9	4.39 ± 2.13	
Grade	1^a^	125	29.2	3.43 ± 2.03	8.110 (<0.001)
	2^b^	171	40.0	4.19 ± 2.07	a < b, c
	3^c^	132	30.8	4.46 ± 2.27	
Family structure	Double-parent family	362	84.6	3.96 ± 2.07	−2.152 (0.032)
	Single-parent family/No parent family	66	15.4	4.58 ± 2.56	
Employment status of parents	Both working parents	262	61.2	4.05 ± 2.05	−0.044 (0.965)
	Single working parent/No working parent	166	38.8	4.06 ± 2.32	
Subjective economic level	High	101	23.6	3.76 ± 1.92	1.980 (0.139)
	Intermediate	306	71.5	4.10 ± 2.17	
	Low	21	4.9	4.71 ± 2.86	
Subjective health status	Healthy	362	84.6	4.00 ± 2.10	−1.09 (0.276)
	Unhealthy	66	15.4	4.32 ± 2.45	
Smartphone ownership period (year)	<2	43	10.0	3.52 ± 2.23	3.841 (0.010)
	2~3	46	10.7	3.35 ± 2.21	
	3~4	65	15.2	3.88 ± 2.00	
	≥4	274	64.1	4.29 ± 2.14	

Among the types of smartphone use, videos, movies, and TV were the most prevalent (4.46 ± 0.79 points), while adolescents' smartphone use for educational video was the least prevalent (2.48 ± 1.07 points). FoMO was measured at 2.15 ± 1.07 points. The score for parental support was 3.86 ± 0.73 points, support from teachers was measured at 3.62 ± 0.68 points, and peer support was scored at 3.85 ± 0.72 points. The smartphone screen time was 4.05 ± 2.16 h (Table 2).

**Table 2:** Intrapersonal, interpersonal factors, and smartphone screen time (*N* = 428).

**Variables**	**M ±SD**	**Range**	**Skewness**	**Kurtosis**
Messengers	4.11 ± 0.95	1~5	−1.061	0.756
Social media	3.59 ± 1.44	1~5	−0.626	−0.998
Games	3.53 ± 1.19	1~5	−0.496	−0.619
Videos/movies/TV	4.46 ± 0.79	1~5	−1.782	3.568
Information-searching/web-surfing	3.31 ± 1.09	1~5	−0.304	−0.586
Educational videos	2.48 ± 1.07	1~5	0.322	−0.502
FoMO	2.15 ± 1.07	1~5	0.627	−0.406
Parental support	3.86 ± 0.73	1.13~5.0	−0.522	0.196
Teachers support	3.62 ± 0.68	1.50~5.0	−0.011	0.191
Peers support	3.85 ± 0.72	1.50~5.0	−0.504	0.237
Smartphone screen time	4.05 ± 2.16	1~12	1.176	1.760

*FoMO, Fear of Missing Out*.

### Differences in Smartphone Screen Time by Socio-Demographic Characteristics

Differences in smartphone screen time by socio-demographic characteristics are shown in Table 1. The significant differences in smartphone screen time were gender (*t* = −5.031, *p* < 0.001), age (*F* = 7.931, *p* < 0.001), grade (*F* = 8.110, *p* < 0.001), and family structure (*t* = −2.152, *p* = 0.032). Female students spent significantly more time on their smartphones, compared to their male counterparts, while students aged 13 and above used their smartphones significantly longer than 12-year-old students.

### Relationship Between Intrapersonal Factors, Interpersonal Factors, and Smartphone Screen Time

The analysis of correlations between major variables is shown in Table 3. The smartphone screen time showed significantly positive correlation with smartphone use for messengers (*r* = 0.165, *p* = 0.001), social media (*r* = 0.266, *p* < 0.001), video/movies/TV (*r* = 0.159, *p* = 0.001), and FoMO (*r* = 0.372, *p* < 0.001). Meanwhile, the smartphone screen time showed significantly negative correlation with smartphone use for educational videos (*r* = −0.229, *p* < 0.001), parental support (*r* = −0.314, *p* < 0.001) and peer support (*r* = −0.121, *p* = 0.012).

**Table 3:** Correlation among intrapersonal, interpersonal factors, and smartphone screen time (*N* = 428).

	**1**	**2**	**3**	**4**	**5**	**6**	**7**	**8**	**9**	**10**
1. Messengers										
2. Social media	0.446^***^									
3. Games	0.045	−0.113^*^								
4. Videos/movies/TV	0.199^***^	0.171^***^	0.319^**^							
5. Information-searching/web-surfing	0.200^***^	0.129^**^	0.029	0.095^*^						
6. Educational videos	0.053	−0.075	0.014	−0.004	0.315^***^					
7. FoMO	0.087	0.128^**^	−0.094	−0.023	−0.021	−0.098^*^				
8. Parental support	0.08	0.004	0.046	0.032	0.184^***^	0.156^**^	−0.281^***^			
9. Teachers support	0.158^**^	0.073	−0.024	0.031	0.05	0.061	−0.120^*^	0.311^***^		
10. Peers support	0.292^***^	0.238^***^	−0.134^**^	0.005	0.063	0.07	−0.282^***^	0.339^***^	0.267^***^	
11. Smartphone screen time	0.165^**^	0.266^***^	0.056	0.159^**^	−0.066	−0.229^***^	0.372^***^	−0.314^***^	−0.073	−0.121^*^

* *p < 0.05*,

** *p < 0.01, and*

*** *p < 0.001*.

*1, messengers; 2, social media; 3, games; 4, videos/movies/TV; 5, information-searching/web-surfing; 6, educational videos; 7, FoMO; 8, Parental support; 9, teachers support; 10, peers support*.

### Factors Associated With Smartphone Screen Time

The analysis result of factors associated with smartphone screen time is explained in Table 4. Before the analysis, some of the socio-demographic characteristics that generated significant differences in smartphone screen time—gender, age, and family structure—were taken as dummy variables. As variance inflation factors in this study ranged from 1 to 10, there was no issue of multicollinearity among the independent variables. Considering the skewness and kurtosis of all the independent variables, a normal distribution was confirmed.

**Table 4:** Hierarchical regression results to identify factors associated with smartphone screen time (*N* = 428).

**Characteristics**	**Model I**			**Model II**			**Model III**		
	**β**	***t***	***p***	**β**	***t***	***p***	**β**	***t***	***p***
Messengers	0.035	0.712	0.477	0.053	1.084	0.279	0.062	1.281	0.201
Social media	0.154	3.103	0.002	0.158	3.233	0.001	0.155	3.228	0.001
Games	0.128	2.617	0.009	0.130	2.713	0.007	0.140	2.943	0.003
Videos/movies/TV	0.104	2.274	0.023	0.100	2.246	0.025	0.084	1.915	0.056
Information-searching/web-surfing	−0.055	−1.213	0.226	−0.023	−0.515	0.607	−0.025	−0.557	0.578
Educational videos	−0.160	−3.582	0.000	−0.144	−3.288	0.001	−0.130	−2.965	0.003
FoMO	0.296	6.577	0.000	0.233	4.903	0.000	0.227	4.827	0.000
Parental support				−0.219	−4.681	0.000	−0.212	−4.585	0.000
Teachers support				0.034	0.773	0.440	0.021	0.478	0.633
Peers support				−0.030	−0.607	0.544	−0.031	−0.648	0.517
Games* Peers support							−0.099	−2.379	0.018
Educational videos* Parental support							0.096	2.342	0.020
Videos/movies/TV* Parental support							−0.084	−2.053	0.041
*F* (*p*)	15.648 (<0.001)			14.648 (<0.001)			13.395 (<0.001)		
*R* ^2^		0.273			0.316			0.343	
*R*^2^ change		0.273			0.043			0.027	

*Adjusted socio-demographic variables (gender, age, and family structure)*.

As Table 4 shows, social media (β = 0.155), game (β = 0.140), and FoMO (β = 0.227) were positively associated with smartphone screen time. Conversely, educational videos (β = −0.130) and parental support (β = −0.212) were negatively associated with it.

### Testing for the Moderation Model

**Figure 2:**
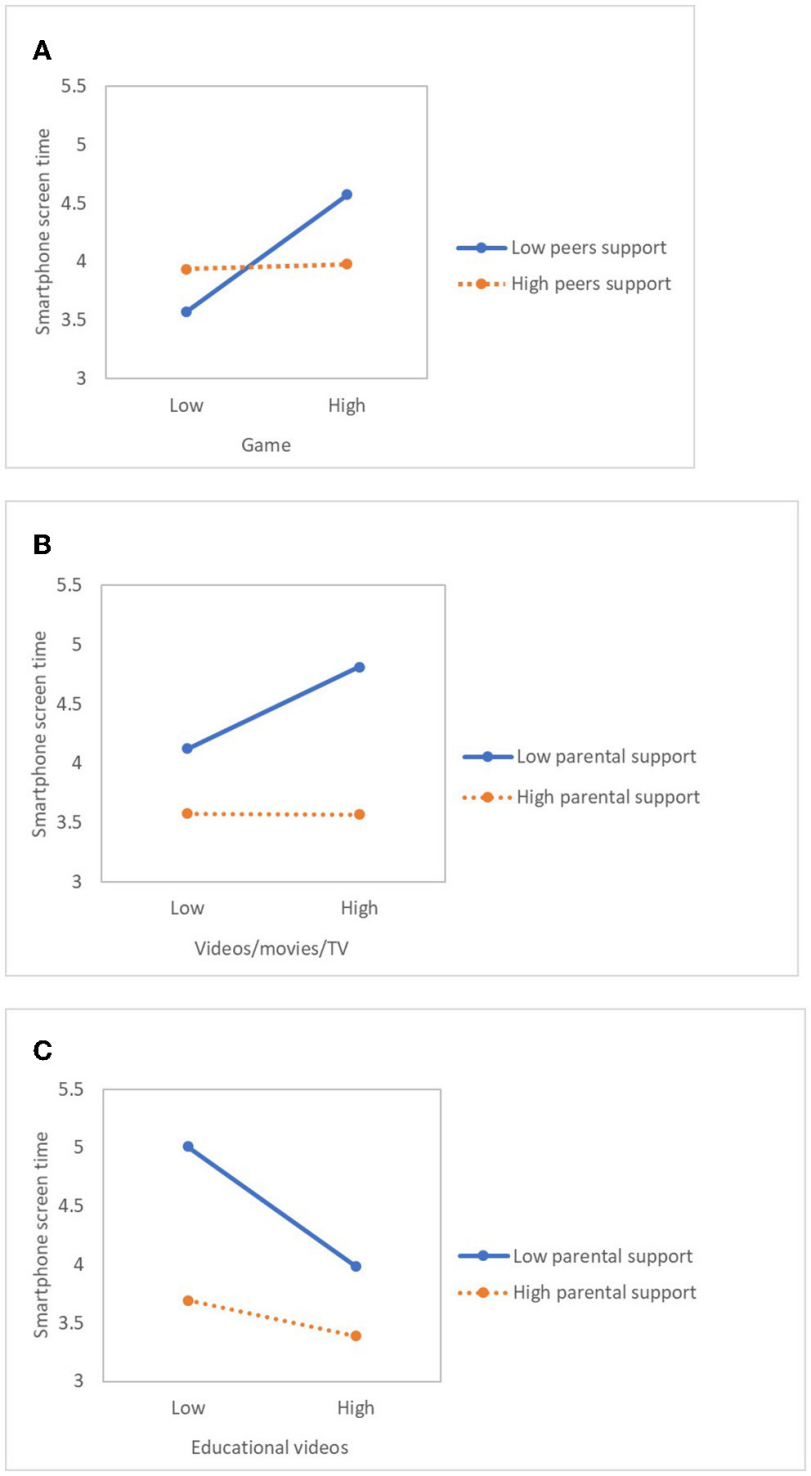
Moderating effect of support from parents and peers. **(A)** Moderating effect of peer support on the relationship between game and smartphone screen time. **(B)** Moderating effect of parental support on the relationship between Videos/movies/TV and smartphone screen time. **(C)** Moderating effect of parental support on the relationship between educational videos and smartphone screen time.

As Table 4 shows, there was a significant interaction between games and peers support (β = −0.099), educational videos and parental support (β = 0.096), and videos/movies/TV and parental support (β = −0.084). For descriptive purposes, the study plotted the regression of smartphone screen time on games at high and low levels of peers support (see Figure 2). This implies that games pose a greater impact on smartphone use for the group with low peer support, compared to their counterpart. It also means that for the group with low parental support, videos/movies/TV and educational videos have a greater influence on their smartphone use.

## Discussion

Based on the ecological model (16), this study aimed to investigate the factors associated with smartphone usage behaviour among middle school students and to devise a strategy to prevent problematic smartphone use. The types of smartphone use, and FoMO were selected as intrapersonal factors that have an impact on the smartphone usage behaviour among middle school students, while parental support, teachers support, and peer support were chosen as interpersonal factors. The smartphone screen time was chosen as a dependent variable. Intrapersonal and interpersonal factors were verified to investigate which factors are related to the smartphone screen time of middle school students.

The daily smartphone screen time was about 4 h, which was more than in other countries and the recommended time for adolescents (2 h or less per day) (39, 40). As evidence is accumulating, linking long use of smartphones and screen-based sedentary behaviour with poorer health outcomes among adolescents, there is a need for measures to reduce the time spent on smartphones by them (1, 2, 39, 40).

In the final models containing intrapersonal and interpersonal factors, significant variables that had relations with the smartphone screen time of middle school students were social media, game, educational videos, FoMO and parental support.

In this study, if adolescents used their smartphones frequently for social media, game among many other purposes of smartphone use, they tended to have a longer smartphone screen time. Subsequently, if their smartphone was used frequently for educational video content, their screen time was shorter than their peers. Such results are similar to the conclusions presented by prior studies, which show that time spent on social media and game is linked to more chances of smartphone addiction, whereas smartphone use for educational purposes is inversely related to problematic smartphone use (6, 22, 23). Social media is not just to maintain or expand one's social relationships, but it is also to express oneself (41). In particular, social media serves as a tool for adolescents to maintain their peer relationships and to increase social capitals. Meanwhile, social media is also related to the difficulties adolescents have with their interpersonal relationships in real life and is associated with problematic smartphone use (1, 6, 22, 23).

Adolescents in various stressful situations can relieve their inner tensions by enjoying entertainment content such as social media/game through their smartphones so that their psychological and physical stress can be mitigated. However, long hours of smartphone use can cause problematic smartphone use, eventually leading to lower academic achievements (22, 42, 43). Therefore, counsellors, educators, and school healthcare managers should understand the purpose of smartphone use among adolescents and devise education and various strategies to help them properly enjoy such content.

In this study, participants with a higher level of FoMO were found to be spending more time on their smartphones, which is a similar result to the findings of prior studies that say, higher levels of FoMO are related to problematic smartphone use (26–28). The peer group of adolescents is an important role model for sociability and behavioural development, and serves as a social group just like family or school (44). Adolescents are heavily dependent on smartphones to have dynamic interactions with their peers and to not be alienated from their peer group (8). Adolescents with unstable peer relationships tend to be more dependent on smartphones so as to decrease a sense of alienation and anxiety (45). Adolescents heavily use their smartphones to feel a sense of belonging with their friends. The recent previous studies have shown that one's level of FoMO is affected by psychological factors such as self-esteem, depression, anxiety or stress (25, 27, 46). Also, a multifaceted approach is required to help adolescents to have psychological well-being.

This study identified that adolescents who received low levels of support from their parents spent longer time on their smartphone. This is consistent with another research finding that adolescents with more parental support have lower smartphone screen time (25, 47, 48). When adolescents recognise support from their parents, they experience a high level of psychological well-being with improved self-control, ultimately decreasing risks of problematic smartphone use (19). When parents deliver a clear message to ban excessive use of smartphones at home, applying needed rules, the smartphone screen time of adolescents tends to decrease (48). All this implies that the parental support has direct and indirect effects on the smartphone behaviour of the children.

In short, adolescents' smartphone screen time is associated with intrapersonal and interpersonal factors as explained in the ecological model. According to the results of this study, elements such as types of smartphone use, FoMO and parental support should be considered when one designs intervention programmes to help adolescents with their smartphone use. As there are various purposes of using smartphones for middle school students, they should be encouraged to use the mobile devices for beneficial purposes such as educational video or searching for information, while they are instructed to manage their screen time properly for social media and game.

In this study, FoMO was associated with middle school students' screen time the most. Adolescents spend much time on their smartphones out of fear that they might be alienated from the culture of their peer group (8). In an effort to maintain good interpersonal relationships, adolescents could use social media or messengers such as KakaoTalk (mobile messaging app widely used in Korea). They also should learn how to build and maintain their in-person relationships as well. When they have friendships where they feel close to and share feelings with their peers, they are expected to spend less time on smartphones alone or to be obsessed with mobile devices from the FoMO. The next strongest determinant to the smartphone screen time of adolescents was parental support. When their emotional needs were fulfilled by parental support and affection, their dependency on smartphones decreased (49). As positive support by parents has an impact on how much adolescents use their smartphone per day, parenting education should be offered so that parents can help their children to not overuse their smartphone.

A significant interaction was found between games and peer support, educational video, videos/movies/TV, and parental support. It implies that for the group with low peer support, games have a greater influence on smartphone use. It was also found in research on peer relationships and smartphone dependency that middle school students tended to be spending less time on their smartphones when their peer relationships improved (8, 50). In a similar context, another study (51) suggested that programmes to improve peer relationships among adolescents can reduce time spent on smartphones playing games. This shows that middle school students experience psychological maladjustment when their peer relationship is unfavourable, which can lead to the overuse of smartphones to play games (8, 50, 51). Such studies imply the possibility that worsened peer relationships can be followed by smartphone dependency.

For the group with low parental support, educational videos and videos/movies/TV were found to be more influencing their smartphone screen time. Also, those with high parental support were found to be controlling smartphone use, using it for utility and fun-seeking purposes (6, 7, 24). Parents' democratic childcare in which parents affectionately take care of their child and provide appropriate guidelines for behaviours were shown to be reducing the child's smartphone addiction (24). Adolescents create an image of themselves, whether good or not, based on the emotional support from their parents, and build behavioural habits (48). Therefore, there is a need for an educational programme that reinforces the parent-child bond, as it is closely associated with problematic behaviours of adolescents.

The significance of this study is as follows: First, intrapersonal and interpersonal factors were investigated in a multifaceted way to understand factors associated with smartphone screen time of middle school students. Second, as this study finds out how much middle school students use their smartphones is related to FoMO, parental support, peer support, it provides a theoretical basis to devise intervention programmes that are focused on those factors. Consequently, this study is expected to serve as basic data to devise problematic smartphone use prevention programmes for middle school students.

Meanwhile, there is some limitation of the study. First, it is a cross-sectional study which analyses relevant elements related to the smartphone screen time of middle school students. While the study identifies whether or not the variables are associated with their smartphone use, the causal relationships between variables cannot be explained. Second, as the study used particular variables of the ecological model, the research cannot explain if other factors such as the community or political factors are related with smartphone use as well. Third, the study recruited participants from limited regions. Thus, there is limitation to generalise the results. It is proposed for further studies to investigate the relations between political factors of ecological model and smartphone use of adolescents in broader regions.

## Conclusion

With the aim of presenting empirical evidence to prevent problematic smartphone use of middle school students, the study investigated the factors associated with smartphone screen time of Korean middle school students, mainly focusing on intrapersonal and interpersonal factors, based on the ecological model by McLeroy et al. (16).

The results showed that the smartphone screen time of middle school students in Korea was related to social media, games, educational videos, FoMO, and parental support. Additionally, parental and peer support moderated the association between the types of smartphone use and smartphone screen time. Therefore, multifaceted approach education programmes should be developed and applied based on the study results to help middle school students properly use their smartphones.

## Data Availability Statement

The raw data supporting the conclusions of this article will be made available by the authors, without undue reservation.

## Ethics Statement

The studies involving human participants were reviewed and approved by Woosuk University, the Institutional Review Board. Written informed consent to participate in this study was provided by the participants' legal guardian/next of kin.

## Author Contributions

J-HK and H-YS contributed to conception and design of the study, performed the statistical analysis, and wrote the first draft of the manuscript. J-HK organised the database. All authors contributed to manuscript revision, read, and approved the submitted version.

## Conflict of Interest

The authors declare that the research was conducted in the absence of any commercial or financial relationships that could be construed as a potential conflict of interest.

## Publisher's Note

All claims expressed in this article are solely those of the authors and do not necessarily represent those of their affiliated organizations, or those of the publisher, the editors and the reviewers. Any product that may be evaluated in this article, or claim that may be made by its manufacturer, is not guaranteed or endorsed by the publisher.
